# C14DM Ablation Leads to Reduced Tolerance to Plasma Membrane Stress and Increased Drug Sensitivity in *Leishmania major*

**DOI:** 10.3390/ijms26178473

**Published:** 2025-08-31

**Authors:** Samrat Moitra, Sumit Mukherjee, Veronica L. Hernandez, Kai Zhang

**Affiliations:** Department of Biological Sciences, Texas Tech University, Lubbock, TX 79409, USA; samrat.moitra21@gmail.com (S.M.); sumit.mukherjee@txstate.edu (S.M.); velherna@utmb.edu (V.L.H.)

**Keywords:** *Leishmania*, sterol, synergy

## Abstract

Sterol biosynthesis is crucial for the function of biological membranes and an important target for anti-protozoan/anti-fungal drugs. In the trypanosomatid parasite *Leishmania major*, the deletion of sterol C14-demethylase (C14DM) results in hypersensitivity to heat, increased plasma membrane fluidity, profound mitochondrial dysfunctions, and reduced virulence in mice. In this study, we show that C14DM-null mutants are defective in their tolerance to membrane-disrupting agents and osmotic stress and their ability to form autophagosomes. In addition, C14DM-null mutants exhibit a heightened sensitivity to anti-trypanosomatid drugs including antimony, ethidium bromide, and pentamidine. The combination of itraconazole (a C14DM antagonist) and pentamidine synergistically inhibits the growth of *Leishmania* parasites. These findings reveal new insight into the roles of sterol synthesis in protozoan pathogens and highlight the potential of using drug combinations to achieve better treatment outcomes.

## 1. Introduction

Trypanosomatid parasites of the genus *Leishmania* cause a spectrum of diseases from localized skins lesions to potentially lethal infections of the liver, spleen, and bone marrow. During their life cycle, these protozoans transition between flagellated promastigotes in sandflies and non-flagellated amastigotes in macrophages. To survive in the vector and mammalian hosts, *Leishmania* parasites must withstand challenges from digestive enzymes, plasma membrane stress, nutrient restriction, and adaptive immunity [1]. Understanding the mechanisms *Leishmania* utilize to counter various stresses may lead to the development of better treatments. This is significant because current drugs are plagued with high toxicity and low efficacy and resistance is on the rise [2].

Sterols are essential components of the plasma membrane regulating membrane fluidity and permeability. Inhibitors of sterol biosynthesis have been explored as drugs against fungi and trypanosomatids [3,4,5]. Among the 20 or so enzymes involved in sterol synthesis, a prominent drug target is the cytochrome P450-dependent sterol 14α-demethylase (C14DM). In *Leishmania major*, genetic or chemical ablation of C14DM leads to the accumulation of 14-methylated sterol intermediates and the loss of ergostane-based sterols; the C14DM-null mutants (*c14dm^−^*) show an increased plasma membrane fluidity, hindered mitochondrial respiration, reduced translation efficiency, and hypersensitivity to heat and glucose restriction; yet, despite these defects, *c14dm^−^* mutants are viable as promastigotes in cultures and as amastigotes in mice, although with slower growth rates [6,7,8]. In *Leishmania donovani*, an early study using the plasmid complementation method failed to generate the chromosomal C14DM-null mutant [9], yet a later report using the CRISPR-Cas9-based gene deletion approach successfully produced the mutant [10], questioning the essentiality of C14DM in *L. donovani*. These and other findings suggest that the inhibition of C14DM alone may not be sufficient to clear a *Leishmania* infection [11,12,13]. Meanwhile, the anti-*Leishmania* effects of C14DM inhibitors such as azoles may be enhanced if they are used in combination with other chemicals including those targeting the weaknesses displayed by *c14dm^−^* [7,14,15]. To this end, it is important to reveal new roles of C14DM in *Leishmania* biology and directly test the effects of drug combinations containing C14DM inhibitors.

In this study, we evaluated the sensitivity of *L. major c14dm^−^* mutants to plasma membrane stress and explored the potential of using inhibitor combinations against several *Leishmania* species. Results suggest that the fitness costs of C14DM inhibition can be exploited for better anti-*Leishmania* therapy.

## 2. Results

### 2.1. C14dm^−^ Mutants Are Hypersensitive to Triton X-100, Dimethyl Sulfoxide (DMSO), and Osmotic Changes

**Figure 1:**
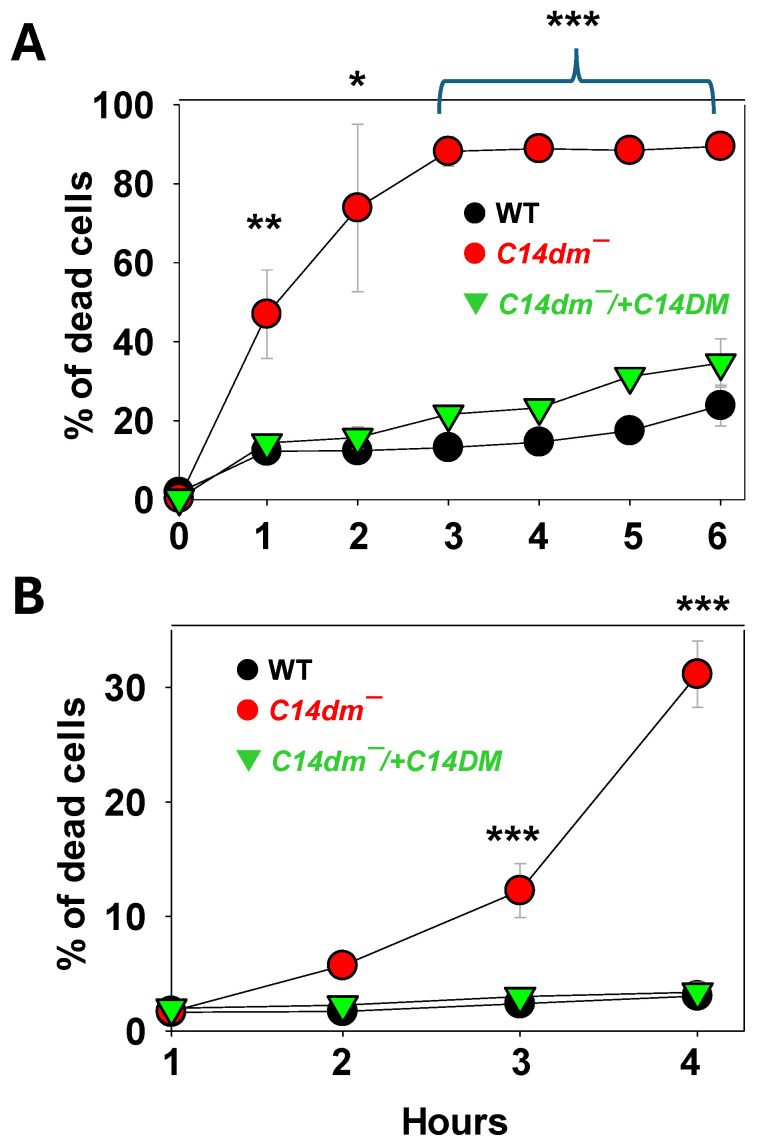
*C14dm^−^* mutants are hypersensitive to membrane-perturbation agents. Log-phase promastigotes of WT, *c14dm^−^*, and *c14dm^−^*/+C14DM were treated with 0.0125% of Triton X-100 (**A**) or 0.9% of DMSO (**B**). Percentages of dead cells were determined hourly by flow cytometry after propidium iodide staining. Error bars represent standard deviations from three repeats. Statistical significances were calculated between groups of *c14dm^−^* and WT (***: *p* < 0.001, **: *p* < 0.01, *: *p* < 0.05).

In *L. major*, C14DM inactivation leads to an increased plasma membrane fluidity and failure to retain vital components of lipid rafts such as GP63 [6]. To further explore how changes in sterol composition affect membrane stability, log-phase promastigotes were incubated in complete M199 media containing 0.0125% Triton X-100. Cell survival was monitored by the propidium iodide exclusion assay. As a non-ionic detergent, Triton X-100 can generate small pores in the membrane at low concentrations [16]. After 1 and 3 h incubation, about 50% and 90% of *c14dm^−^* mutants became permeable to propidium iodide (indicating cell death), respectively, in comparison to <30% for the *L. major* wild-type (WT) and the complemented *c14dm^−^*/+C14DM parasites after 6 h of incubation in the same condition (Figure 1A). Similar results were observed when log-phase promastigotes were challenged with 0.9% DMSO, another membrane permeabilization agent, for a period of 1–4 h (Figure 1B). Thus, C14DM inactivation causes hypersensitivity to membrane-disrupting chemicals.

**Figure 2:**
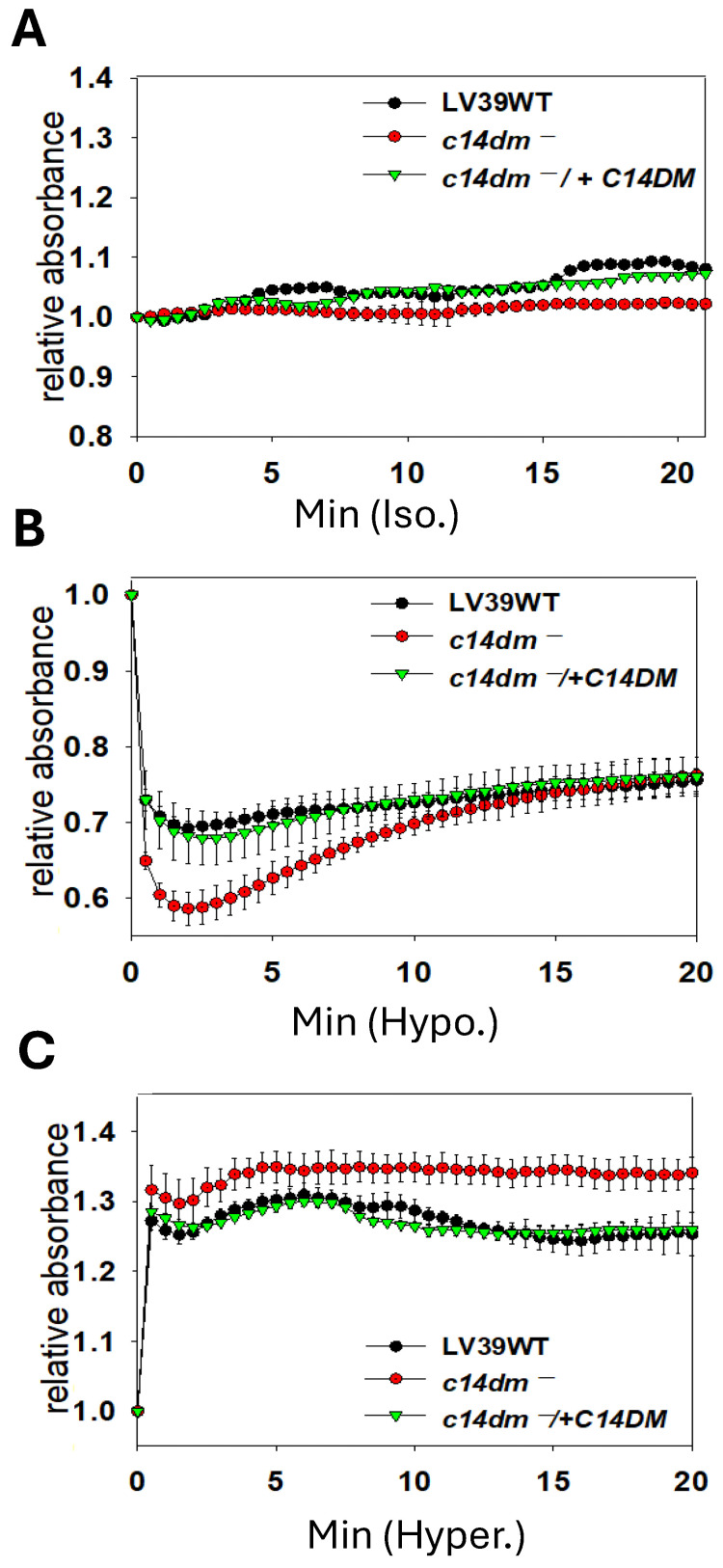
*C14dm^−^* mutants show altered response to osmotic stress. Log-phase promastigotes were resuspended in isotonic ((**A**), 300 mOsmol/L), hypotonic ((**B**), 150 mOsmol/L), or hypertonic ((**C**), 600 mOsmol/L) solutions, as described in Materials and Methods. Responses to osmotic changes (regulatory volume decrease/increase) were monitored by light-scattering measurements at 550 nm using a Biotek plate reader (every 30 s for 60 min). Error bars represent standard deviations from four repeats.

Along the same lines, we examined whether C14DM plays a role in osmoregulation. *L. major* WT, *c14dm*^−^, and *c14dm*^−^/+*C14DM* promastigotes were cultivated in their regular media (complete 1 × M199) [17] and then subjected to isotonic (Figure 2A), hypotonic (0.5 X, Figure 2B), or hypertonic (2.0 X, Figure 2C) conditions. Their cell volume change was measured by light-scattering spectrophotometry, as previously described [18]. This method monitors the swelling, shrinking, and recovery of cells in response to osmotic changes. Under hypotonic stress, *c14dm*^−^ mutants showed more severe swelling than WT and *c14dm*^−^/+*C14DM* parasites after 2–3 min, as indicated by reduced light absorbance. After 15–20 min, *c14dm*^−^ mutants recovered to similar levels as WT and *c14dm*^−^/+*C14DM* (Figure 2B). With hypertonic challenge, *c14dm*^−^ mutants shrank more than WT and *c14dm*^−^/+*C14DM* cells (Figure 2C) and were unable to restore their volume to the same degree during the period of the experiment (up to 60 min). Therefore, *c14dm*^−^ mutants are hypersensitive to osmotic stress.

### 2.2. C14dm^−^ Mutants Show Hypersensitivity to Pseudomonas aeruginosa Spent Medium and Pyocyanin

**Figure 3:**
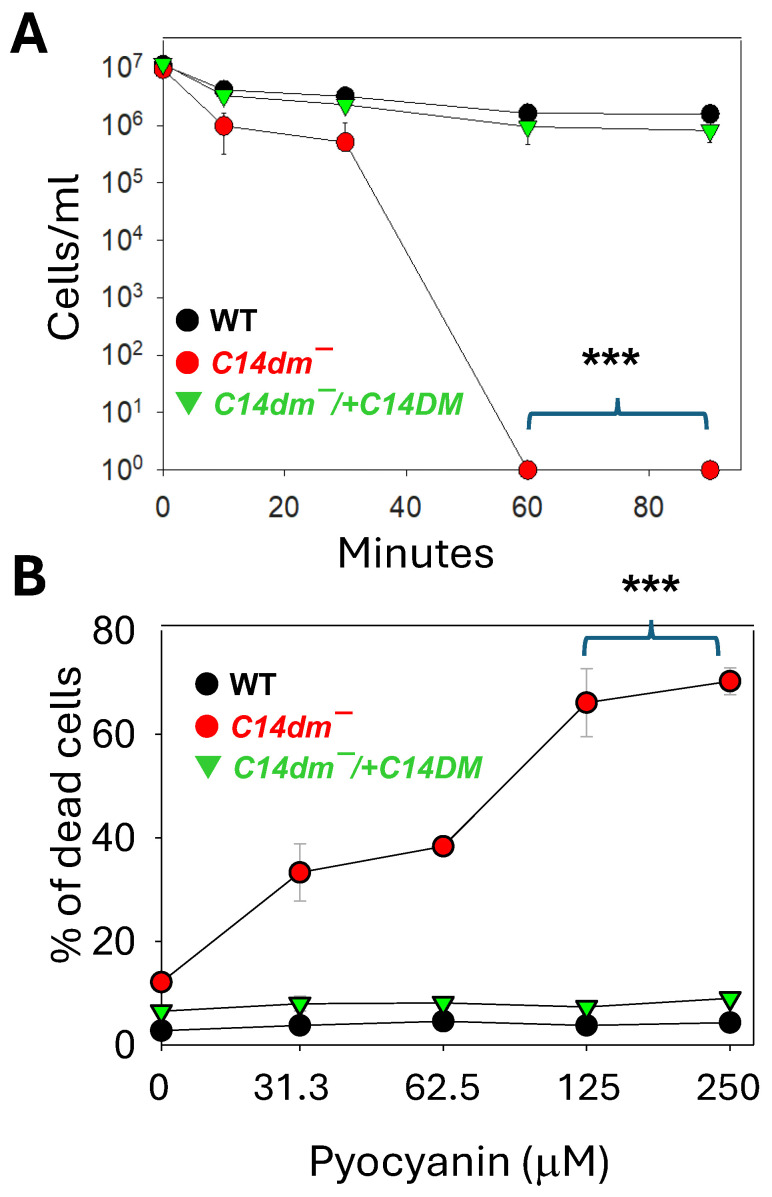
*C14dm^−^* mutants are hypersensitive to *Pseudomonas aeruginosa* products. Log-phase promastigotes of WT, *c14dm^−^*, and *c14dm^−^*/+*C14DM* were incubated in Pseudomonas aeruginosa PA14 spent medium (**A**) or various concentrations of pyocyanin (**B**), as described in Materials and Methods. In (**A**), the remaining cells/mL were monitored at 0–90 min post-incubation. In (**B**), percentages of dead cells were determined hourly by flow cytometry after 24 h. Error bars represent standard deviations from four repeats. Statistical significances were calculated between groups of *c14dm^−^* and WT (***: *p* < 0.001).

*Leishmania* protozoans are vector-borne parasites which form complex interactions with bacteria both in the sandfly and the mammalian host’s skin. Various bacterial species may affect *Leishmania* development and disease progression through competition for nutrients, production of toxins, and modulation of the host’s immune response [19,20,21,22]. Here, we examined the response of *L. major* promastigotes to the metabolites of *Pseudomonas aeruginosa*, a Gram-negative opportunistic bacterium that has been identified from multiple sandfly species and reported to complicate cutaneous leishmaniasis [23,24,25]. To do so, the *P. aeruginosa* strain PA14 (a common laboratory reference strain) was cultivated in a complete 1 × M199 medium (the same medium for growing *Leishmania* promastigotes) until the OD600 reached 3.0; the spent medium was isolated by centrifugation and filtration; and log-phase parasites were incubated in the PA14 spent medium, and their survival was monitored by direct cell counting. As summarized in Figure 3A, the PA14 spent medium is highly toxic to *L. major* promastigotes. For WT and *c14dm*^−^/+*C14DM*, ~20% of the parasites remained viable after 90 min. In comparison, *c14dm*^−^ mutants were even more sensitive: after 10 min, only ~10% were viable and after 60 min, all of them were lysed. The potent leishmanicidal activity of the PA14 spent medium is likely due to the plethora of cytolytic toxins produced by *P. aeruginosa* [26,27].

We also explored whether *C14DM* is involved in the tolerance to other cytotoxic products from *P. aeruginosa*, such as pyocyanin, a water-soluble, phenazine-derived pigment metabolite capable of inducing reactive oxygen species (ROS) in target cells [28,29]. When grown in the presence of pyocyanin (0–250 μM), *c14dm*^−^ mutants showed a significant and dose-dependent level of cell death (33–70%) after 24 h. WT and add-back parasites were not affected by the pyocyanin treatment at the same concentration range (Figure 3B). Together, these findings demonstrate the importance of sterol synthesis in *Leishmania* resistance to *P. aeruginosa* products.

### 2.3. C14dm^−^ Mutants Show Autophagy Defects

**Figure 4:**
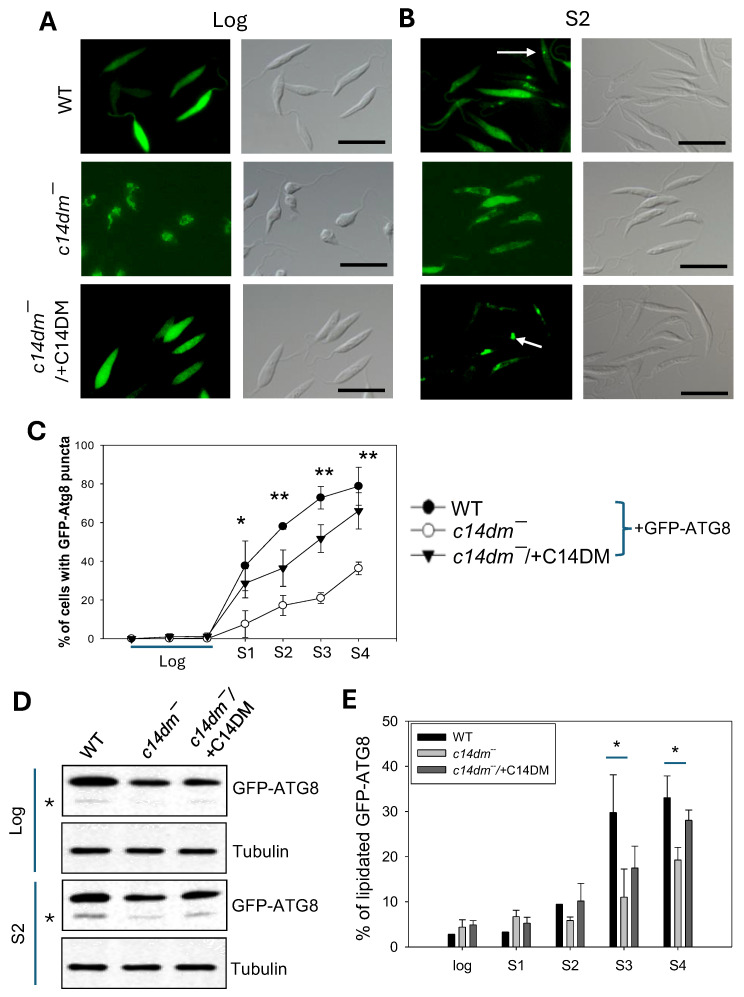
*C14dm^−^* mutants show defects in autophagy. Promastigotes of WT, *c14dm^−^*, and *c14dm^−^*/+*C14DM* containing GFP-ATG8 were cultivated from log phase to stationary phase (day 1-day 4 as S1-S4). (**A**–**C**) The formation of GFP-ATG8 puncta representing autophagosomes (examples were marked by arrows) was monitored by fluorescence microscopy. Representative fluorescence images and DIC images are shown in (**A**) (log) and (**B**) (S2). Quantified results are shown in (**C**). Scale bars in (**A**,**B**): 10 μm. (**D**,**E**) Lipidation of GFP-ATG8 was determined by Western blot using an anti-GFP antibody (in (**D**), asterisks represent lipidated GFP-ATG8). The anti-tubulin antibody was used as a loading control. (**E**) Quantitation of Western blots showing the percentages of lipidated GFP-ATG8. Error bars represent standard deviations from three repeats. More microscopy and Western blot images are included in Supplemental Figures S1 and S2. Statistical significances were calculated between groups of *c14dm^−^* and WT (**: *p* < 0.01, *: *p* < 0.05).

Consistent with their mitochondrial deficiencies, the *c14dm*^−^ mutants are more dependent on glucose uptake and glycolysis than WT parasites for energy production [7]. Consequently, they are extremely vulnerable to glucose starvation. Here, we investigate whether the capacity to carry out autophagy may also contribute to the compromised response of *c14dm*^−^ mutants to starvation by introducing GFP-ATG8 (a known marker for macroautophagy) into promastigotes and then monitoring its cellular location and lipidation [30,31]. As WT promastigotes grew in culture, GFP-ATG8 transitioned from mostly cytoplasmic in the log phase to 40–80% punctate structures in the stationary phase, indicating an increased formation of autophagosomes as parasites encountered reduced nutrient levels in the culture (Figure 4A–C and Supplemental Figures S1 and S2) [30,31]. In *c14dm*^−^, GFP-ATG8 displayed an uneven, tubular, intracellular distribution during the log phase and only 8–36% of cells showed GFP-ATG8 puncta in the stationary phase (Figure 4A–C and Supplemental Figures S1 and S2). These anomalies were largely reversed to WT-levels in the *c14dm*^−^/+*C14DM* add-back parasites. We also examined the lipidation of GFP-ATG8 by Western blot. While log-phase parasites mainly contained the non-lipidated GFP-ATG8, stationary-phase parasites had both non-lipidated and fast-migrating-lipidated GFP-ATG8 (GFP-ATG8-PE). Compared to WT cells, *c14dm*^−^ mutants had less GFP-ATG8-PE during the stationary phase (Figure 4D,E and Supplemental Figures S1 and S2). The formation of GFP-ATG8 puncta and the lipidation of GFP-ATG8 are considered autophagy markers in *Leishmania* promastigotes under starvation conditions or during differentiation [30,31,32]. Thus, our findings suggest that *c14dm*^−^ mutants are deficient in autophagy.

### 2.4. C14dm^−^ Parasites Show Increased Sensitivity to Antimony and Ethidium Bromide (EtBr)

**Figure 5:**
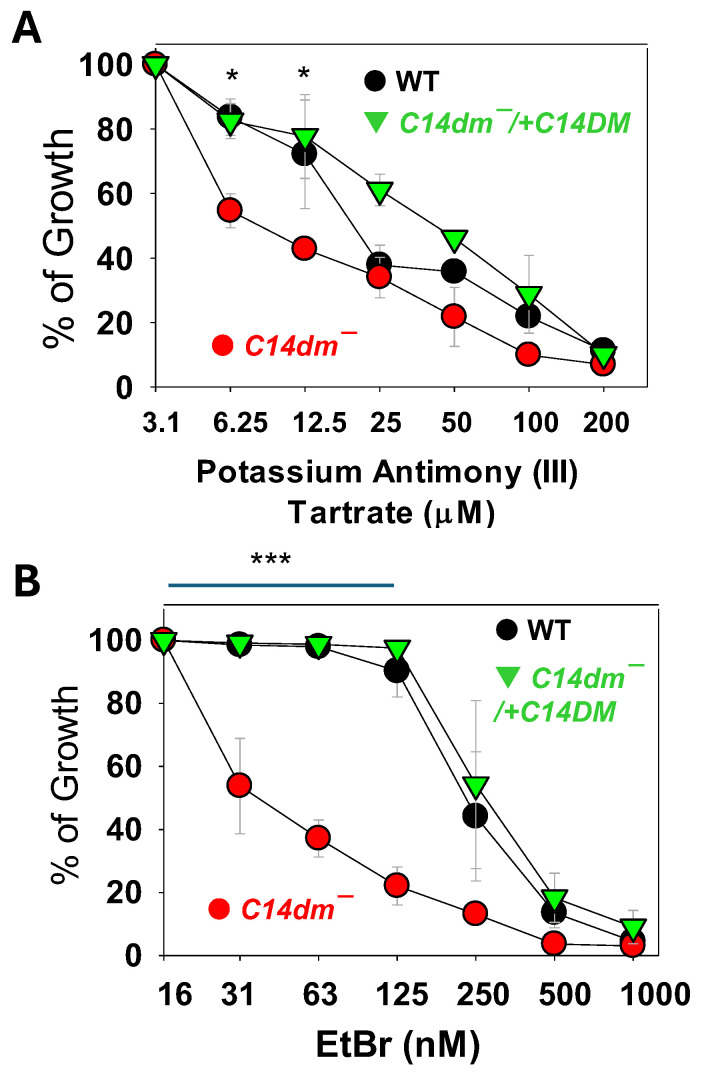
*C14dm^−^* mutants are hypersensitive to antimony III and ethidium bromide (EtBr)**.** Log-phase promastigotes of WT, *c14dm^−^*, and *c14dm^−^*/+*C14DM* were cultivated in the presence of potassium antimony (III) tartrate (**A**) or EtBr (**B**). Cell growth was monitored after 48 h and compared to cultures grown in the absence of drugs. Error bars represent standard deviations from three repeats. Statistical significances were calculated between groups of *c14dm^−^* and WT (***: *p* < 0.001, *: *p* < 0.05).

The hypersensitivity of *c14dm*^−^ mutants to various stress conditions including heat, starvation, membrane perturbation, and osmolality change prompts us to investigate whether such hypersensitivity can be exploited to develop better antileishmanial agents. We first tested the efficacy of potassium antimony (III) tartrate, a classic drug used to treat leishmaniasis and trypanosomiasis [2,33]. In the culture, *c14dm*^−^ mutants showed a dose-dependent response with an EC50 of 6.3 ± 0.6 μM, which was significantly lower than WT (EC50: 19.0 ± 1.3 μM) and *c14dm*^−^/+*C14DM* promastigotes (EC50: 39.2 ± 3.0 μM). Similarly, *c14dm*^−^ mutants were much more sensitive to EtBr, which interferes with kDNA replication and causes growth arrests [34,35], than WT and add-back *L. major* promastigotes (Figure 5B; EC50 for *c14dm*^−^: 32 ± 2.4 nM; EC50 for WT; and *c14dm*^−^/+*C14DM*: 250–280 nM).

### 2.5. Synergistic Inhibition of Leishmania mexicana and Leishmania donovani with Pentamidine (PENT) and Itraconazole (ITZ)

In *L. major*, the genetic or chemical ablation of *C14DM* causes an increased susceptibility to PENT, an anti-trypanosomatid agent known to be sequestered in the mitochondria of target cells [7,36]. Here, we tested whether this finding could be extended to other *Leishmania* species.

**Figure 6:**
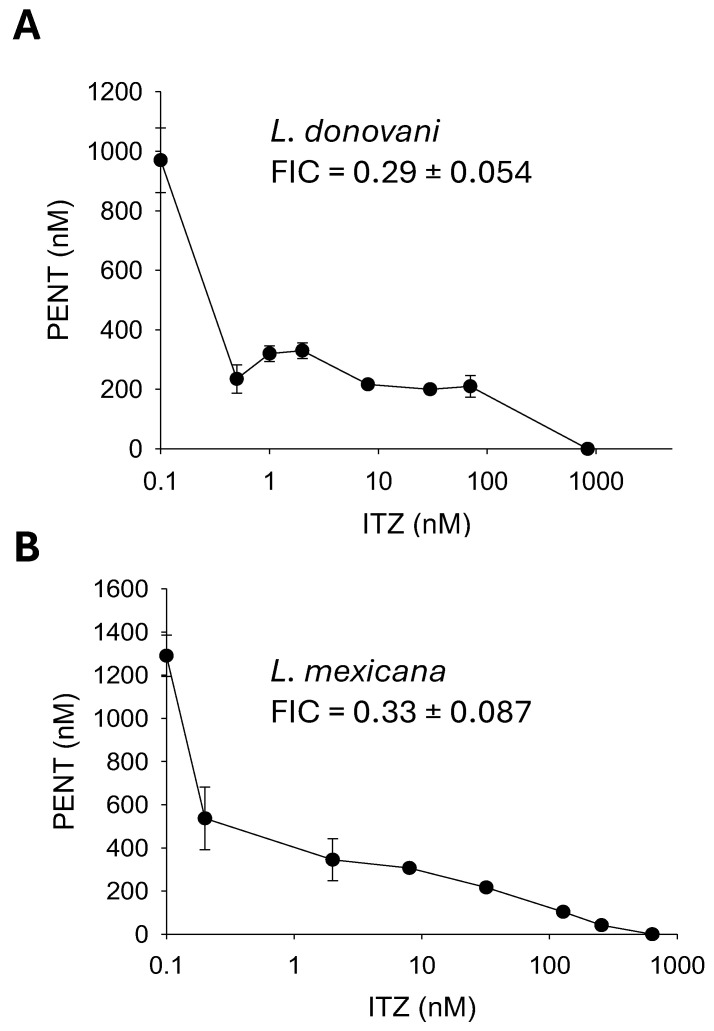
Synergistic inhibition of *Leishmania* promastigotes with itraconazole (ITZ) and pentamidine (PENT). Log-phase promastigotes of *L. donovani* (**A**) or *L. mexicana* (**B**) were cultivated in various concentrations of ITZ (X axis) and PENT (Y axis). EC50 values were determined and plotted in an isobologram. Fractional inhibitory concentrations (FIC) were calculated as described in Materials and Methods (average ± SDs). Error bars represent standard deviations from four repeats.

For *Leishmania donovani* and *Leishmania mexicana* promastigotes, their EC50s to PENT were 1.01 ± 0.039 μM and 1.28 ± 0.099 μM, respectively (Figure 6). To generate *c14dm*^−^ phenocopies, we cultivated cells in the presence of ITZ (0–1 μM), a *C14DM* inhibitor [6]. The EC50s to ITZ for *L. donovani* and *L. mexicana* were 0.84 ± 0.24 μM and 0.64 ± 0.178 μM, respectively (Figure 6). When grown in low concentrations of ITZ (<EC50 values), the sensitivities of *L. donovani* and *L. mexicana* to PENT were determined, and their EC50 values for PENT were plotted in an isobologram (Figure 6). Both *L. donovani* and *L. mexicana* were more susceptible to PENT in the presence of low-dose ITZ, indicating that PENT and ITZ work synergistically [37]. The mean fractional inhibitory concentration (FIC) from seven PENT/ITZ combinations was determined to be 0.29 ± 0.054 for *L. donovani* and 0.33 ± 0.087 for *L. mexicana* (Figure 6) (FIC < 0.5 is considered synergistic) [37].

### 2.6. C14DM Inhibition Enhances Drug Sensitivity in L. mexicana Axenic Amastigotes

**Figure 7:**
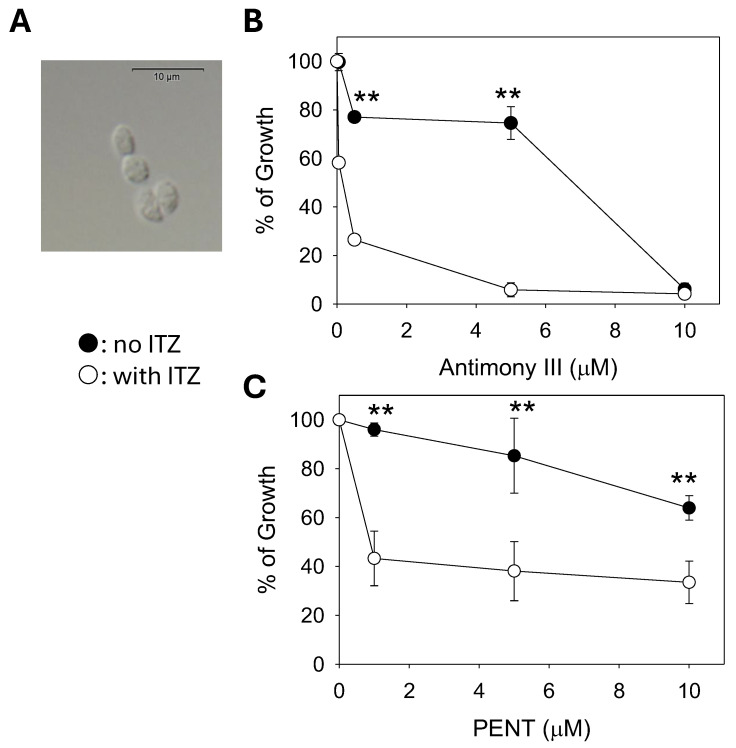
Chemical inhibition of *C14DM* makes *L. mexicana* axenic amastigotes hypersensitive to antimony III and pentamidine (PENT). Axenic amastigotes of *L. mexicana* (**A**) cultivated in the absence or presence of 50 nM of ITZ were treated with various concentrations of potassium antimony III tartrate (**B**) or PENT (**C**). Cell growth was monitored after 72 h in comparison to cells growing in the absence of ITZ. Error bars represent standard deviations from four repeats. Statistical significances were calculated between groups of no ITZ and with ITZ (**: *p* < 0.01).

Finally, we examined the impact of C14DM inhibition on the drug susceptibility of *L. mexicana* axenic amastigotes, which mimic intracellular amastigotes [38]. When cultivated in the presence of 50 nM of ITZ, axenic amastigotes of *L. mexicana* displayed a heightened sensitivity to potassium antimony III tartrate (EC50: 98 ± 10 nM) and PENT (EC50: 1.0 ± 0.06 μM); both values are significantly lower than those without ITZ (EC50 for antimony III without ITZ: 8.3 ± 0.44 μM; EC50 for PENT without ITZ: >12 μM); thus, *C14DM* inhibition by ITZ may enhance drug sensitivity in *Leishmania* amastigotes (Figure 7).

## 3. Discussion

C14DM catalyzes the heme-dependent oxidative removal of the C-14 methyl group from sterol intermediates, a critical step in sterol biosynthesis. C14DM deletion in *L. major* results in a complete loss of ergostane-based sterols, which is largely replaced by 14-methylated sterol intermediates. This drastic change in sterol composition leads to increased plasma membrane fluidity, extreme vulnerability to heat, and significant mitochondrial abnormalities leading to the accumulation of reactive oxygen species (ROS) and impairment in respiration [6,7]. In this study, we further explored the stress response defects in *c14dm^−^* mutants and investigated the potential of exploiting these defects to improve drug efficacy. First, we found *c14dm^−^* mutants to be highly sensitive to membrane perturbation agents, including Triton X-100 (0.0125%) and DMSO (0.9%), as a large portion of them died within hours while WT and add-back parasites were mostly alive (Figure 1). In addition, *c14dm^−^* cells showed more pronounced volume changes than WT parasites under hypo- and hyper-osmolarity stress (Figure 2). These findings suggest that the accumulation of 14-methylated sterols, mainly 14-methylfecosterol, renders the plasma membrane more permeable and less stable.

We also tested the role of C14DM in the parasite’s response to *Pseudomonas aeruginosa* products. During the sandfly stage, *Leishmania* resides in the midgut, which harbors a diverse and dynamic microbiota [23,39,40]. *Leishmania* interacts with the sandfly midgut microbiome through competition for nutrients and space; parasites also encounter toxins and other bacterial metabolites; and the content and abundance of the microbiome can either facilitate or hinder *Leishmania* survival and development [41,42,43]. During the mammalian stage, *Leishmania* and bacteria coinfections have been reported to exacerbate skin lesions [21,24,25,44]. Here, we used *P. aeruginosa* as a model to evaluate the impact of sterol synthesis on *Leishmania*–bacteria interactions. As shown in Figure 3A, the spent medium from *P. aeruginosa* PA14 had potent leishmanicidal activity, as ~80% of WT *L. major* promastigotes were lysed with 60 min. This is likely due to the cytotoxic substances secreted by *P. aeruginosa* including apoptosis-inducing toxins (e.g., ToxA, ExoS, and ExoT) and cytolytic toxins (e.g., ExlA, CTX, and ExoU) [26,27]. Significantly, the loss of C14DM led to a much more rapid cell death, indicating that the plasma membrane defects in *c14dm^−^* mutants enhanced the binding and/or uptake of *P. aeruginosa* toxins. *C14dm^−^* mutants also exhibited an increased sensitivity to pyocyanin, a pigmented secondary metabolite from *P. aeruginosa* which targets the electron transport chain causing the accumulation of ROS [28,29]. It is possible that pyocyanin exacerbates the mitochondrial injury in *c14dm^−^*, leading to increased levels of cell death.

Additionally, we examined whether *C14DM* deletion may affect the parasite’s ability to carry out autophagy by monitoring the clustering (puncta formation) and lipidation of GFP-ATG8 in stationary-phase cultures. Autophagy is not only important for the recycling of cellular components and nutrients but also the transition from replicating promastigotes to infective metacyclics and then intracellular amastigotes [32,45]. The autophagy defects exhibited by *c14dm^−^* may contribute to their compromised virulence and tolerance to starvation stress (Figure 4 and Supplemental Figures S1 and S2). It is not clear how the perturbation of sterol synthesis affects the autophagy process, and future studies may focus on the roles of sterols in vesicular trafficking and nutrient sensing.

Our previous study on *c14dm^−^* has revealed the increased sensitivity of these mutants to pentamidine (PENT), a drug known to be sequestered into the mitochondria of *Leishmania* in a membrane potential-dependent manner to exert its activity [7,36]. Here, we discovered a similar hyper-susceptibility of *c14dm^−^* to EtBr and antimony (Sb III) (Figure 5). As a DNA-intercalating agent, EtBr impairs both kinetoplast DNA and nuclear DNA replication [35]. The targets of antimony-based drugs include the trypanothione reductase/trypanothione system, DNA topoisomerase I, and host immunity, leading to elevated ROS stress, DNA replication defects, and an activated immune response [46,47,48]. The high membrane permeability in *c14dm^−^* may increase drug uptake and target binding in *c14dm^−^*. Additionally, these drugs may exacerbate the existing mitochondrial and ROS stress in *c14dm^−^*, resulting in more pronounced cell death or growth inhibition.

Like *c14dm^−^*, *L. major* WT promastigotes grown in the presence of ITZ (a potent inhibitor of *C14DM*) [6] are highly susceptible to PENT [7]. Here, we observed similar findings with *L. donovani* and *L. mexicana*. When used in combination, PENT and ITZ inhibited the growth of these parasites at a greater efficacy than PENT/ITZ alone (Figure 6). By plotting the EC50 values of inhibitor combinations in an isobologram, we revealed a synergistic interaction between PENT and ITZ (instead of additive or antagonistic interactions) (Figure 6) [37]. Finally, the chemical inhibition of *C14DM* by ITZ also caused hypersensitivity to antimony III and PENT in the axenic amastigotes of *L. mexicana*, raising the possibility that such a combination strategy may be viable in vivo (Figure 7).

Other studies have demonstrated that inhibition of multiple targets (within or outside of sterol biosynthesis) can result in improved efficacy in treating leishmaniasis [49,50]. Both combinational therapy and multi-target drug candidates have been explored in this avenue [49,50]. Similar strategies may be applied to diseases caused by other trypanosomatids or fungi that share similar sterol synthesis pathways to *Leishmania*.

In summary, we revealed new insights into the plasma membrane defects in *C14DM*-null mutants, and the potential of using inhibitor combinations to improve the efficacy of current antileishmanial drugs. Future work will explore how lipid metabolism affects *Leishmania* development in the sandfly, and the potential of a synergistic drug combination in the mammalian host, which could result in improved efficacy and reduced side effects.

## 4. Materials and Methods

### 4.1. Materials

Pyocyanin, potassium antimony III tartrate, ethidium bromide (EtBr), itraconazole (ITZ), and pentamidine (PENT) were purchased from Sigma-Aldrich Co (St. Louis, MO, USA). Stock solutions for these chemicals were prepared in sterile phosphate-buffered saline (PBS) or DMSO (for ITZ and PENT) and stored in aliquots at –20 °C. Rabbit anti-GFP antibody was purchased from Abcam (Waltham, MA, USA). All other reagents were purchased from Thermo Fisher Scientific (Waltham, MA, USA) unless specified.

### 4.2. Leishmania Culture

*L. major* LV39 clone 5 wild-type (WT) (Rho/SU/59/P), *c14dm^−^* (*C14DM*-null mutant), and *c14dm^−^*/+*C14DM* (the complemented or add-back line) promastigotes were cultivated at 27 °C in complete M199 media (pH 7.4) with 10% fetal bovine serum and additional supplements, as previously described [6,17]. The same medium and condition were used to culture *Leishmania donovani* strain 1S2D (MHOM/SD/62/1S-CL2D) and *Leishmania mexicana* M379 (MNYC/BZ/62/M379) promastigotes. *L. mexicana* axenic amastigotes were cultured using an amastigote medium based on the *Drosophila* Schneider’s medium, supplemented with 20% fetal bovine serum and 0.0015% hemin (pH 5.4) in vented flasks in a humidified 32 °C/5% CO_2_ incubator [38,51]. To monitor cell growth over time, culture densities were determined using a hemacytometer. Cell viability was measured by flow cytometry after staining with 0.5 µg/mL of propidium iodide using an Attune Acoustic Flow Cytometer (Waltham, MA, USA) [6].

### 4.3. To Determine the Effects of Membrane-Perturbing Agents, P. aeruginosa PA14-Conditioned Medium, Pyocyanin, and Chemical Inhibitors

To test the effect of membrane-disrupting agents, *L. major* promastigotes were cultivated to 2.0 × 10^6^ cells/mL in complete 1 × M199 medium and treated with Triton X-100 (final concentration: 0.0125%) or DMSO (final concentration: 0.9%). Cell viability was measured at various time points by flow cytometry after staining with propidium iodide. We titrated the concentration ranges of Triton X-100 (0–0.1%) and DMSO (0–2%) to determine the optimal concentrations that reveal the difference between WT and *c14dm^−^*. *P. aeruginosa* PA14-conditioned medium was separated from PA14 bacteria grown in complete 1 × M199 medium (OD600: 2.9–3.0) by centrifugation (8000 g, 10 min), followed by filtration through 0.2-micron filters. Log-phase promastigotes were resuspended in the PA14-conditioned medium at 5.0 × 10^6^ cells/mL, and cell survival was measured by counting the number of promastigotes/mL using a hemocytometer at 0–60 min post-exposure.

To determine sensitivity to pyocyanin, log-phase promastigotes were inoculated in complete 1 × M199 medium at 2.0 × 10^5^ cells/mL and challenged with pyocyanin ranging from 31.25 µM to 250 µM. After incubation at 27 °C for 24 h, cell viability was measured by flow cytometry.

To measure sensitivity to potassium antimony III tartrate, ethidium bromide (EtBr), ITZ, or PENT, log-phase promastigotes were inoculated in complete 1 × M199 medium at 2.0 × 10^5^ cells/mL and exposed to various concentrations of inhibitors. Culture densities were determined using a Beckman Z2 Coulter Counter (Indianapolis, IN, USA) after 48 h. The effective concentrations required to inhibit growth by 50% (EC50s) were determined using cells grown in the absence of inhibitors as controls. Similar assays were performed with *L. mexicana* axenic amastigotes cultivated in the amastigote medium.

### 4.4. Synergy Calculations

To determine if the effect produced by a combination of inhibitors is greater than the sum of the effects produced by each inhibitor alone, a classical isobologram was constructed by plotting the EC50s of drugs that acted either singularly or in combination. Fractional inhibitory concentration (FIC) was calculated as previously described [37]:

FIC = EC50 _XY_/EC50_X_ + EC50 _YX_/EC50_Y_

EC50_X_ is the EC50 value for drug × (PENT) acting alone, and EC50_XY_ is the EC50 of the same drug in the presence of a sub-optimal concentration of drug Y (ITZ). Similarly, EC50_Y_ is the EC50 value for drug Y (ITZ) acting alone, and EC50_YX_ is the EC50 of the same drug in the presence of a sub-optimal concentration of drug × (PENT). If the value of the FIC is ≤0.5, a synergic effect is diagnosed; for 0.5 < FIC ≤ 1, the effects are considered additive; and for FIC > 1.0, the combined effects are considered antagonistic [37].

### 4.5. Response to Osmotic Stress

Log-phase promastigotes were collected by centrifugation (2000 g, 10 min), washed twice with PBS, and resuspended in the isotonic 1 × Iso-Cl buffer (20 mM HEPES pH 7.4, 11 mM glucose, 1 mM CaCl_2_, 0.8 mM MgSO_4_, 137 mM NaCl, 4 mM KCl, 1.5 mM K_2_HPO_4_, and 8.5 mM Na_2_HPO_4_; final osmolarity: 301 mOsmol/L) at 2 × 10^8^ cells/mL. Cell suspensions were distributed into a 96-well plate with 150 μL per well in triplicate. To determine cells’ response to osmotic changes, 150 μL of deionized water or 3 × Iso-Cl buffer was added to each well to induce hypotonic or hypertonic stress. For the isotonic control, 150 μL of 1 × Iso-Cl buffer was added. Relative cell volume changes were assessed by monitoring absorbance at 550 nm by light scattering, where a decrease corresponded to an increase in cell volume [18]. Absorbance was measured every 30 s for 60 min using a Biotek plate reader, where every time point was calculated in the automated kinetic format, and orbital shaking was performed for 5 s after each reading. Absorbance values were normalized to cells under the isotonic condition.

### 4.6. Autophagy Assays and Microscopy

For autophagy studies, the GFP-ATG8 open reading frame was cloned into the pXG-HYG plasmid [31,52] and introduced into *L. major* WT, *c14dm^−^*, or *c14dm^−^*/+*C14DM* promastigotes by electroporation. Transfectants were selected and grown in the presence of 40 μg/mL of hygromycin as WT +*GFP-ATG8*, *c14dm^−^* +*GFP-ATG8*, or *c14dm^−^*/+*C14DM* +*GFP-ATG8*.

To measure the percentage of cells with autophagosomes containing GFP-ATG8, promastigotes were analyzed daily from log phase to late stationary phase by fluorescence microscopy, as previously described [30,31,45], using a BX-51 epifluorescence microscope. At each measuring point, a minimum of 200 cells per group were examined and categorized as having either cytosolic or spotty autophagosomal GFP-ATG8 localization (puncta). In addition, Western blot was performed using an anti-GFP antibody to detect both phosphatidylethanolamine (PE)-conjugated and unconjugated GFP-ATG8 [31]. Signals from Western blot were quantified using a Fuji phosphoimager.

### 4.7. Statistical Analysis

Experimental values in all figures were averaged from three to five independent biological repeats, and error bars represented standard deviations. Differences were assessed by one-way ANOVA (for three or more groups) using the Sigmaplot 13.0 software (Systat Software Inc, San Jose, CA, USA). *P* values indicating statistical significance were grouped in all Figures (***: *p* < 0.001, **: *p* < 0.01, and *: *p* < 0.05).

## 5. Conclusions

In *Leishmania major*, the loss of sterol C14-demethylase (C14DM) leads to a significantly reduced tolerance to membrane perturbation agents and osmotic changes. In addition, C14DM-null mutants are highly sensitive to *Pseudomonas aeruginosa* metabolites and anti-trypanosomatid drugs including antimony, ethidium bromide, and pentamidine. A combination of the C14DM blocker itraconazole and pentamidine synergistically inhibits the growth of *Leishmania* parasites. While these findings were acquired using promastigotes in a culture, future studies will evaluate the potential of applying drug combinations against intracellular amastigotes and assess the role of sterol synthesis in *Leishmania*–vector interactions.

## Data Availability

The original contributions presented in the study are included in the article/supplementary material, further inquiries can be directed to the corresponding author.

## Supplementary Materials

The supporting information can be downloaded at: https://www.mdpi.com/article/10.3390/ijms26178473/s1.
