# Supplementary material for: C14DM Ablation Leads to Reduced Tolerance to Plasma Membrane Stress and Increased Drug Sensitivity in Leishmania major
Source: Int J Mol Sci. 2025 Aug 31;26(17):8473. doi: 10.3390/ijms26178473 (PMC12429082; doi:10.3390/ijms26178473)
Supplement: Supplementary file 1 [file ijms-26-08473-s001.zip › ijms-3825489-supplementary.pdf]

SUPPLEMENTAL FIGURE LEGENDS

Figure S1. *C14dm*$^{-}$ *mutants* show defects in autophagy. (**A**-**D**) Promastigotes of WT (**A**), *c14dm*$^{-}$ (**B**), and *c14dm*$^{-}$/+*C14DM* (**C**) containing GFP-ATG8 were cultivated from log phase to stationary phase (day 1-day 4). GFP-ATG8 puncta representing autophagosomes (examples were marked by arrows) were monitored by fluorescence microscopy and quantified in **D**. Scale bars in **A**-**C**: 10 μm. (**E**) Lipidation of GFP-ATG8 was determined by Western blot using an anti-GFP antibody (left, asterisks mark lipidated GFP-ATG8) or anti-tubulin antibody (right, as loading control). (**F**) Quantitation of Western blots showing the percentages of lipidated GFP-ATG8 (**F**). Error bars represent standard deviations from three repeats. Red rectangles represent panels shown in Figure 4.

Figure S2. Full size Western blot images for Figure 4D and Figure S1E. 1: WT, 2: *c14dm*$^{-}$, 3: *c14dm*$^{-}$/+*C14DM*. Rectangles represent panels shown in Figure 4D and S1E.

Supplemental Figure S1

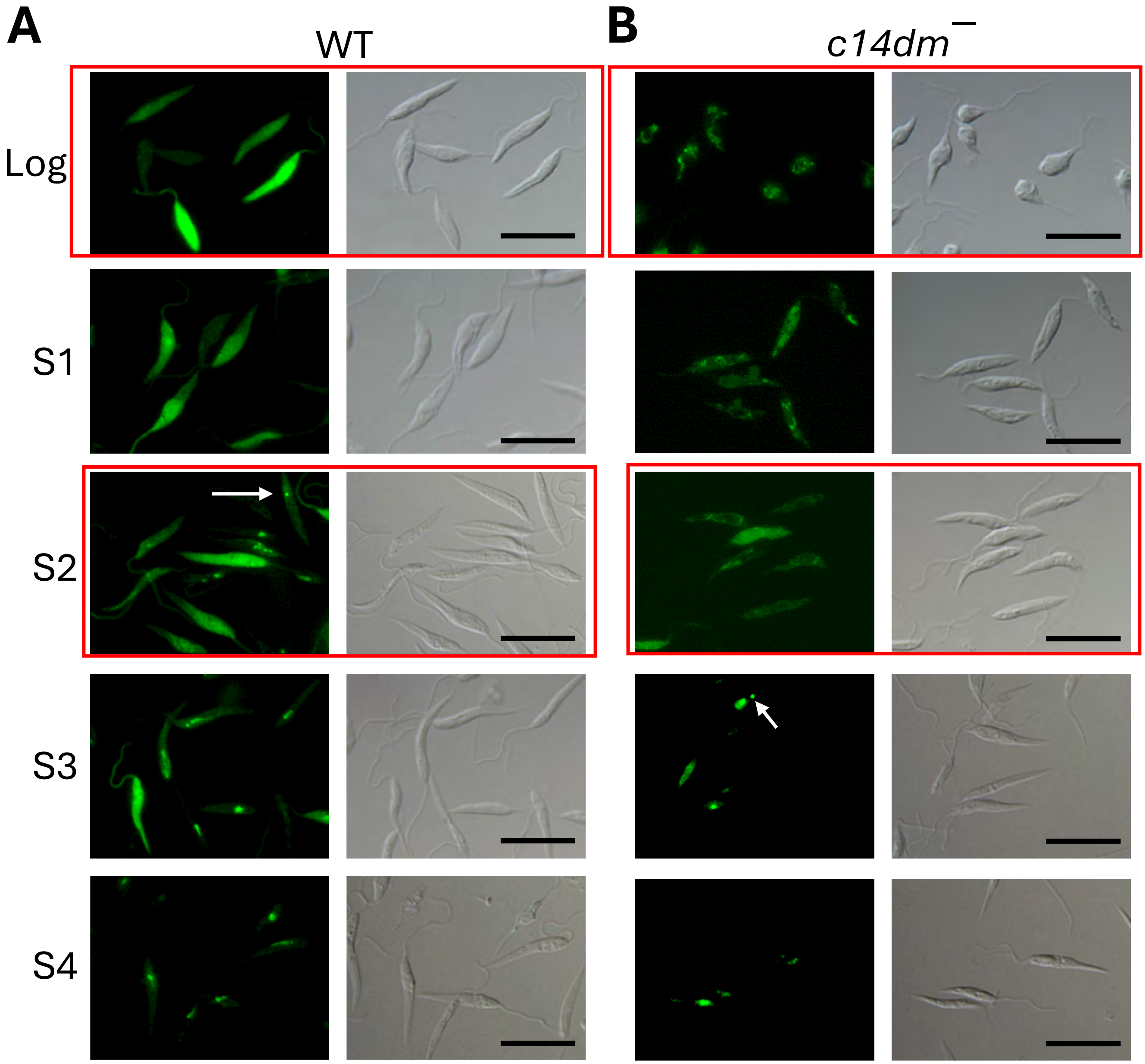

Figure S1. *C14dm⁻mutants* show defects in autophagy. (**A-D**) Promastigotes of WT (**A**), *c14dm⁻* (**B**), and *c14dm⁻/+C14DM* (**C**) containing GFP-ATG8 were cultivated from log phase to stationary phase (day 1-day 4). GFP-ATG8 puncta representing autophagosomes (examples were marked by arrows) were monitored by fluorescence microscopy and quantified in **D**. Scale bars in **A-C**: 10 µm. (**E**) Lipidation of GFP-ATG8 was determined by Western blot using an anti-GFP antibody (left, asterisks mark lipidated GFP-ATG8) or anti-tubulin antibody (right, as loading control). (**F**) Quantitation of Western blots showing the percentages of lipidated GFP-ATG8 (**F**). Error bars represent standard deviations from three repeats. Red rectangles represent panels shown in Figure 4.

Supplemental Figure S1

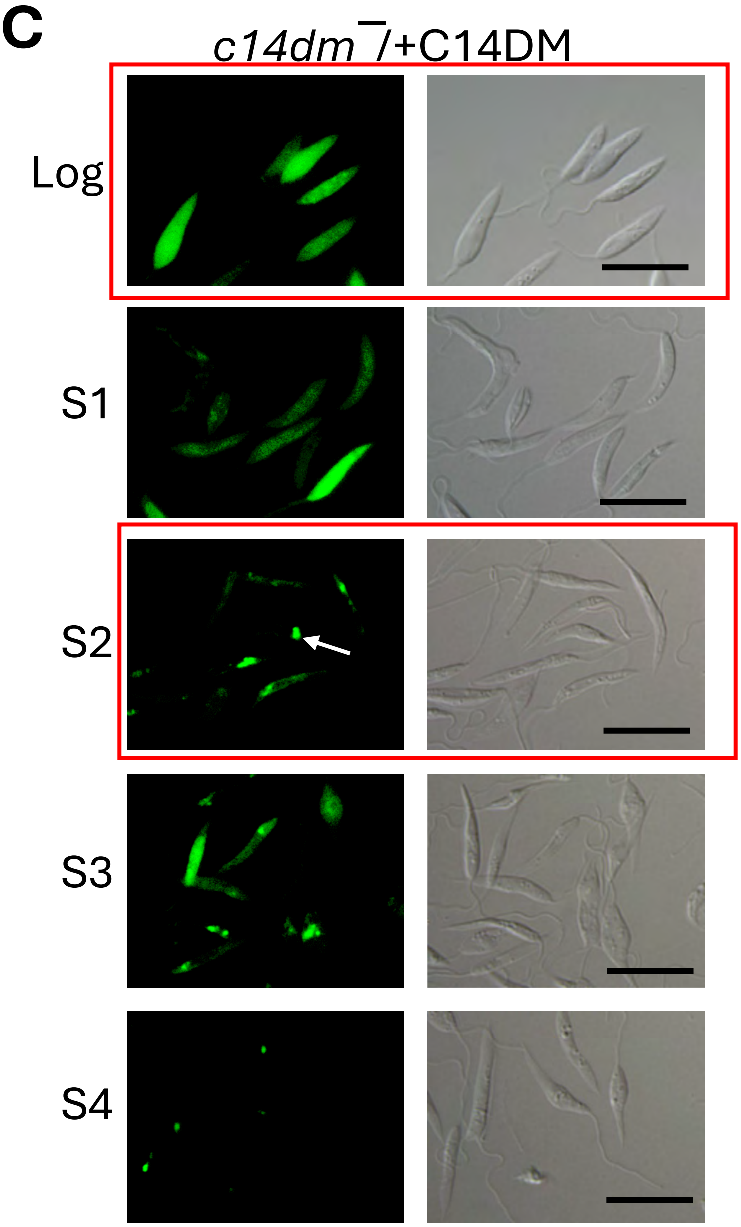

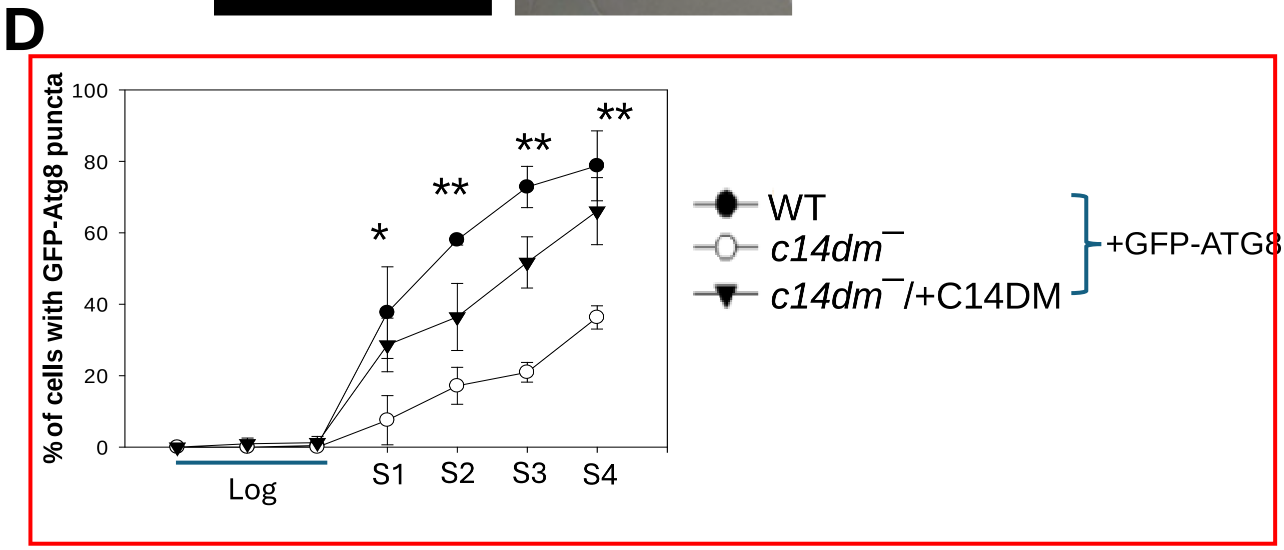

Supplemental Figure S1

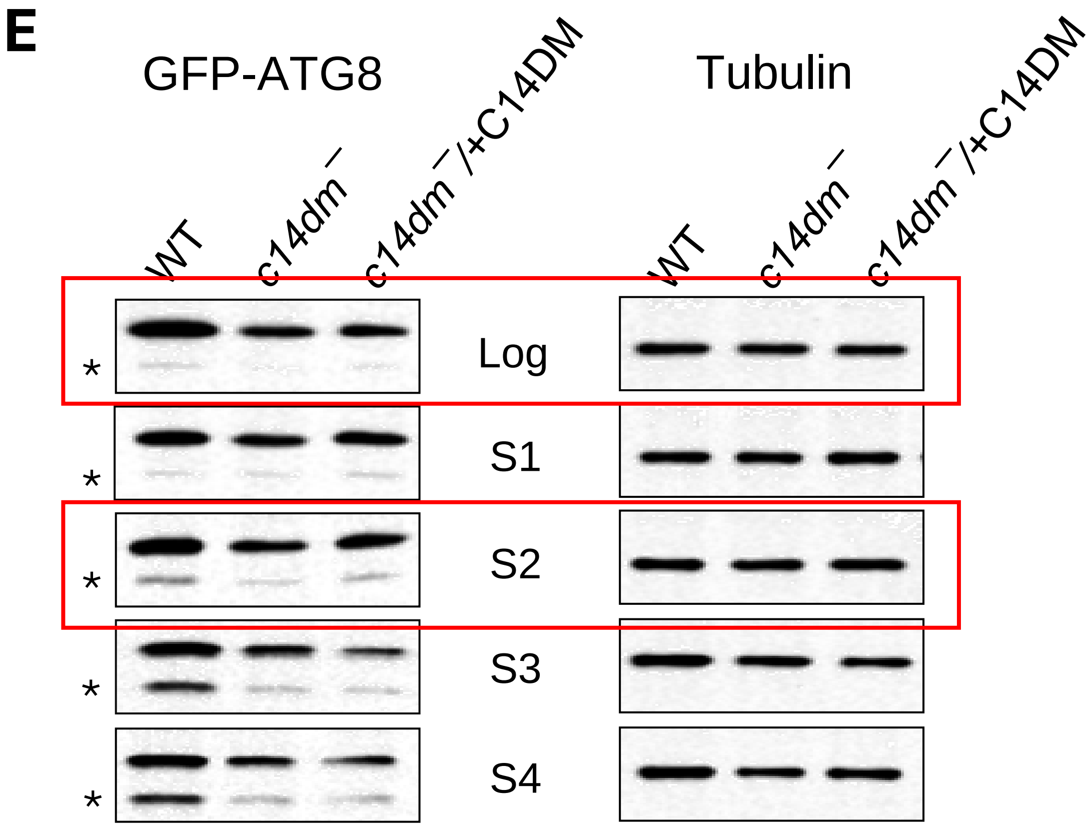

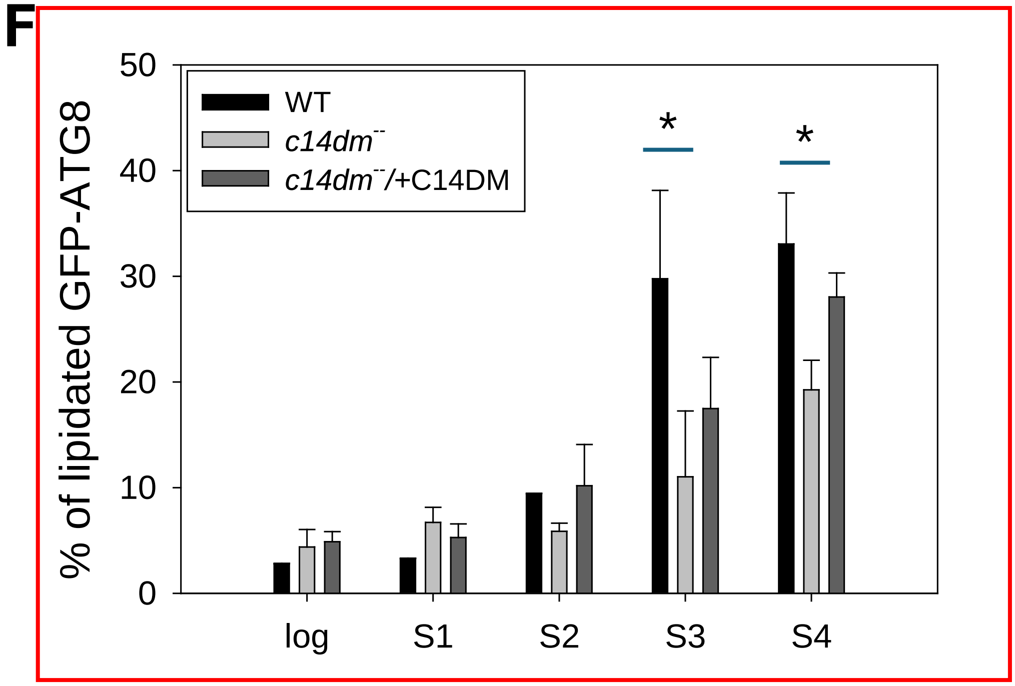

Supplemental Figure S2

For GFP-Atg8 (anti-GFP)

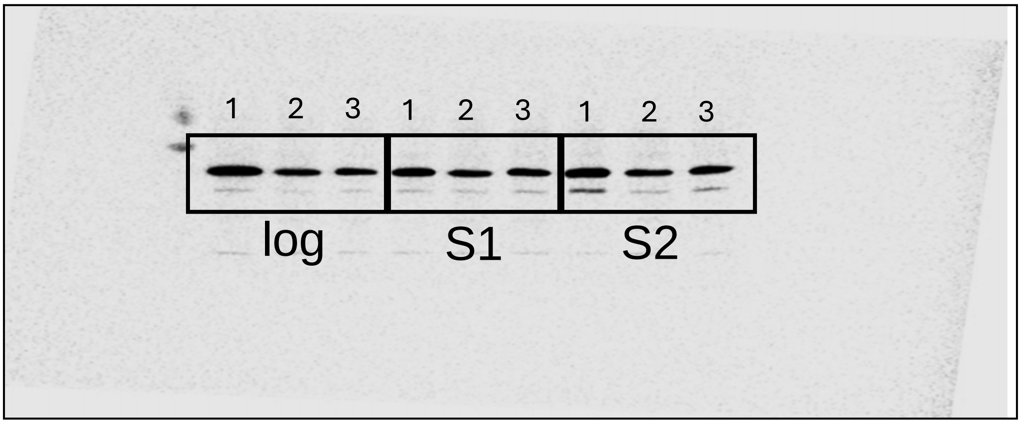

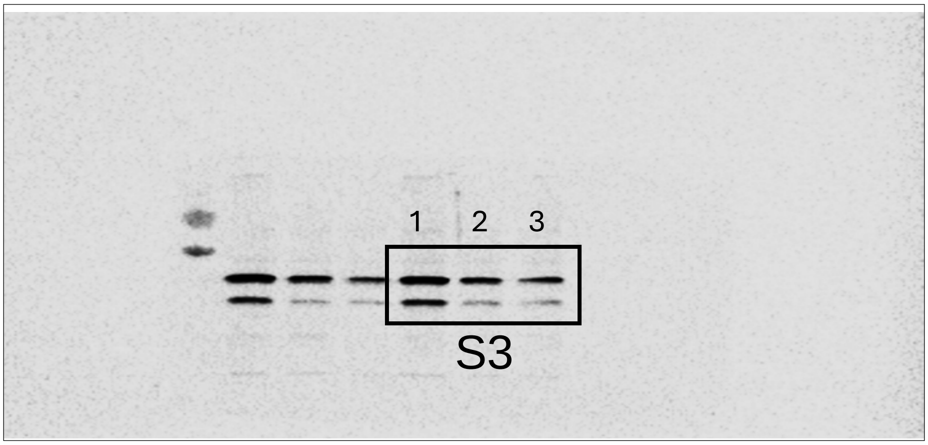

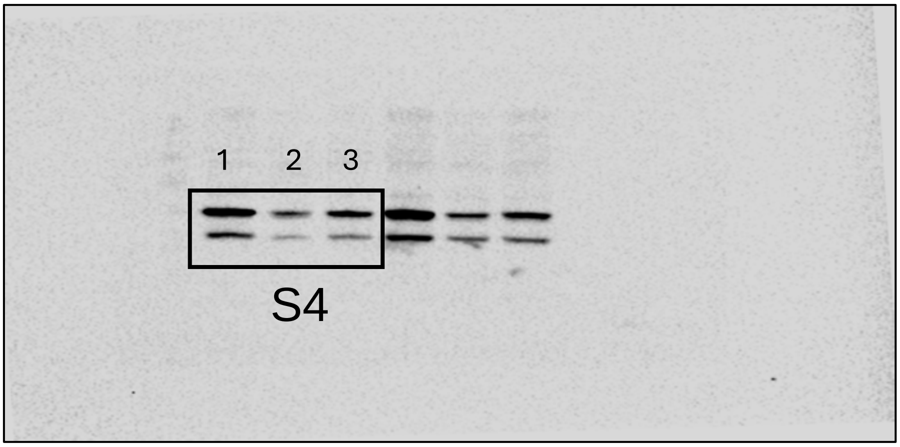

Supplemental Figure S2

Lading control (anti-tubulin)

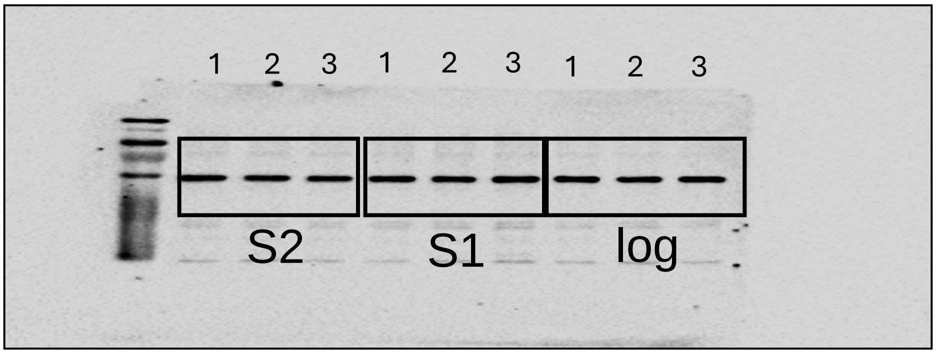

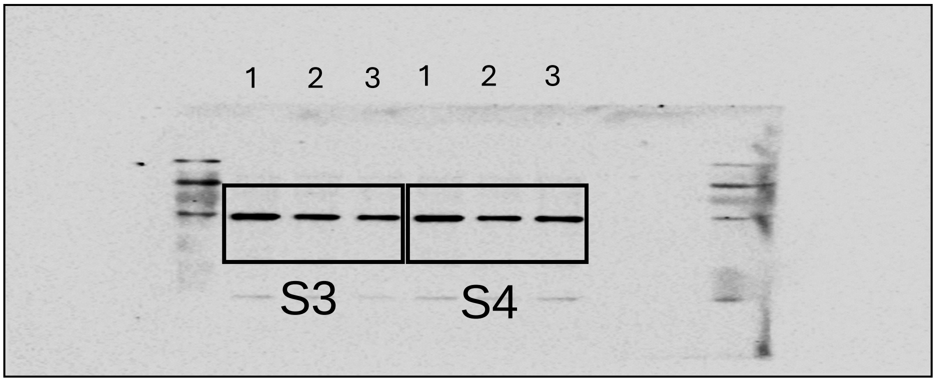

Figure S2. Full size Western blot images for Figure 4D and Figure S1E. 1: WT, 2: *c14dm*$^{-}$, 3: *c14dm*$^{-}$*/+C14DM. R*ectangles represent panels shown in Figure 4D and S1E.
